# Metabolic profile changes in serum of migraine patients detected using ^1^H-NMR spectroscopy

**DOI:** 10.1186/s10194-021-01357-w

**Published:** 2021-11-24

**Authors:** Aster V. E. Harder, Lisanne S. Vijfhuizen, Peter Henneman, Ko Willems van Dijk, Cornelia M. van Duijn, Gisela M. Terwindt, Arn M. J. M. van den Maagdenberg

**Affiliations:** 1 Departments of Human Genetics, Leiden University Medical Centre, Leiden, The Netherlands; 2 Department of Neurology, Leiden University Medical Centre, Leiden, The Netherlands; 3 Department of Clinical Genetics, Genome Diagnostic laboratory, Amsterdam Reproduction & Development research institute, Amsterdam University Medical Centre, Amsterdam, The Netherlands; 4 Einthoven Laboratory for Experimental Vascular Medicine, Leiden University Medical Centre, Leiden, The Netherlands; 5 Department of Internal Medicine, Division of Endocrinology, Leiden University Medical Centre, Leiden, The Netherlands; 6 Department of Epidemiology, Erasmus Medical Centre, Rotterdam, The Netherlands; 7 Nuffield Department of Population Health, Oxford University, Oxford, UK

**Keywords:** ^1^H-NMR spectroscopy, Migraine, Biomarker, Blood, Serum

## Abstract

**Background:**

Migraine is a common brain disorder but reliable diagnostic biomarkers in blood are still lacking. Our aim was to identify, using proton nuclear magnetic resonance (^1^H-NMR) spectroscopy, metabolites in serum that are associated with lifetime and active migraine by comparing metabolic profiles of patients and controls.

**Methods:**

Fasting serum samples from 313 migraine patients and 1512 controls from the Erasmus Rucphen Family (ERF) study were available for ^1^H-NMR spectroscopy. Data was analysed using elastic net regression analysis.

**Results:**

A total of 100 signals representing 49 different metabolites were detected in 289 cases (of which 150 active migraine patients) and 1360 controls. We were able to identify profiles consisting of 6 metabolites predictive for lifetime migraine status and 22 metabolites predictive for active migraine status. We estimated with subsequent regression models that after correction for age, sex, BMI and smoking, the association with the metabolite profile in active migraine remained. Several of the metabolites in this profile are involved in lipid, glucose and amino acid metabolism.

**Conclusion:**

This study indicates that metabolic profiles, based on serum concentrations of several metabolites, including lipids, amino acids and metabolites of glucose metabolism, can distinguish active migraine patients from controls.

**Supplementary Information:**

The online version contains supplementary material available at 10.1186/s10194-021-01357-w.

## Introduction

Migraine is a common multifactorial brain disorder with a lifetime prevalence of 15–20%, causing disability worldwide and a three times higher prevalence in woman compared to men [1, 2]. Migraine is characterized by recurrent episodes of severe often unilateral pulsating headache accompanied by nausea, vomiting and/or photo- and phonophobia lasting for 4–72 h [3]. Although much progress has been made with unravelling its (non) genetic disease mechanisms [4], a diagnosis of migraine is still made by interview and physical examination or questionnaire, as no diagnostic biomarker is available. The lack of biomarkers, for instance in a biofluid such as blood, has also hampered the development of novel treatments.

Metabolomics is an established valuable approach for biomarker identification and has been successful in revealing the metabolic underpinnings of various human diseases [5–10]. Validated biomarkers can greatly improve diagnosis, prognosis and assessing effectivity of treatment of patients, as was already shown for several diseases other than migraine [11, 12]. Various attempts have been made to identify reliable biomarkers (either clinical, genetic, radiological or biochemical) in migraine [13–16], without much success, also not for biochemical studies in blood [17] or cerebrospinal fluid [14]. Especially the identification of metabolites in an easily accessible body fluid such as peripheral blood is urgently needed [18]. When using metabolomics either a targeted approach that typically focuses on one or more related selected pathways of interest or an untargeted approach that aims to simultaneously measure as many metabolites as possible from a biological sample, can be employed. Several biochemical studies in migraine in the past two decades explored the targeted approach by examining a limited number of compounds, such as amino acids [19, 20], inflammatory markers [21–23], vasoactive neuropeptides [24–26], and (cardio) vascular risk factors [27–29], because of their presumed role in migraine pathophysiology. More recently, mainly because of the advent of novel treatment antagonizing calcitonin gene-related peptide (CGRP) or its receptor [30, 31], the field of biomarker research in peripheral blood regained interest [32], with reports of promising possible peripheral biomarkers in migraine [33, 34].

To search for migraine metabolite profiles in serum we used an untargeted, hypothesis-free, approach and performed high-throughput proton nuclear magnetic resonance (^1^H-NMR) spectroscopy. This method allows for a rapid, robust, simultaneous identification and quantification of a variety of metabolites in large numbers of samples [35]. Here we analysed metabolite profiles in serum samples of migraine patients and controls from the Erasmus Rucphen Family population, a large Dutch population-based family study from the Southwest of the Netherlands in which we previously had identified migraine cases [36]. We set out to investigate whether metabolites identified by ^1^H-NMR spectroscopy are associated with migraine by comparing metabolic profiles of migraine patients and controls in a “real-life variation” cohort.

## Material and methods

### Study population

The study included participants from the Erasmus Rucphen Family (ERF) study [37, 38]. This study population is based on a genetically isolated community in the Southwest of the Netherlands. In brief, the ERF study population includes 3465 living descendants of 22 couples that had at least six children baptized in the community church between 1850 and 1900. Hence, study participants were all members of a large extended pedigree and all of European ancestry. All individuals 18 years and older were invited to participate.

### Migraine diagnoses

Migraine was diagnosed using a validated three-stage screening procedure [2], based on International Classification of Headache Disorder formerly ICHD-II, now ICHD-III criteria [3, 39]. Details on the migraine case-finding procedure have been published previously [36]. In short, first, participants filled out a five-item screening questionnaire on headache and aura symptoms. Next, screen-positives completed an additional detailed questionnaire on headache and aura symptoms. Finally, the diagnosis was validated with a telephone interview by a physician trained in headache disorders. Probable migraine patients were excluded. ERF participants who were negative for severe headache and/or migraine based on the aforementioned three-stage screening procedure were included as controls [2, 36]. Samples from participants were collected after overnight fasting.

### ^1^H-NMR spectroscopy metabolite profiling: data processing and quality control

Venous blood samples had been drawn by venipuncture from the median cubital vein from participants of the ERF study after at least 8 h fasting period. Samples were centrifuged at 1000–2000 x g for 10 min at 4 °C and serum was aliquoted in cryovials and stored at − 80 °C until further use. The ^1^H-NMR data were generated as part of a larger project and described by Vaarhorst et al. [40]. All ^1^H-NMR spectroscopy experiments had been acquired on a 600 MHz Bruker Avance II spectrometer (Bruker) equipped with a 5-mm triple resonance inverse (TCI) cryogenic probe head with Z-gradient system and automatic tuning and matching. All experiments were recorded at 310 K. Temperature calibration was done prior to each batch of measurements using the method of Findeisen et al. [41]. The duration of the π/2 pulses were automatically calibrated for each individual sample using a homonuclear-gated nutation experiment on the locked and shimmed samples after automatic tuning and matching of the probe head [42].

Then, stored samples were thawed at 4 °C and mixed by inverting the containers ten times. Samples (300 μL) were mixed with 300 μL 75 mM disodium phosphate buffer in H_2_O/D_2_O (80/20) (pH 7.4), containing 6.15 mM NaN_3_ and 4.64 mM sodium 3-[trimethylsilyl] d4-propionate (TSP), using a Gilson 215 liquid handler in combination with a Bruker SampleTrack system (Bruker, Karlsruhe, Germany). Samples were transferred into 5-mm SampleJet NMR tubes (Bruker) in 96-tube racks using a modified Gilson 215 tube filling station (Gilson, Middleton, WI, USA) and kept at 6 °C on a SampleJet sample changer (Bruker) while queued for acquisition.

For water suppression pre-saturation of the water resonance with an effective field of γB_1_ = 25 Hz was applied during the relaxation delay [43]. J-resolved spectra (JRES) [44] were recorded with a relaxation delay of 2 s and a total of one scan for each increment in the indirect dimension. A data matrix of 40 × 12,288 data points was collected covering a sweep width of 78 × 10,000 Hz. A sine-shaped window function was applied and the data was zero-filled to 256 × 16,384 complex data points prior to Fourier transformation. The resulting data matrix was tilted along the rows by shifting each row (k) by 0.4992*(128-k) points and symmetrised about the central horizontal lines to compensate for the skew of the multiplets in the F1 dimension. For T2-filtered ^1^H-NMR spectra, a standard 1D Carr-Purcell-Meiboom-Gill (CPMG) pulse sequence [45, 46] was used with a relaxation delay of 4 s. A pulse train of 130 refocusing pulses with individual spin echo delays of 0.6 ms were applied resulting in a total T2 filtering delay of 78 ms. A total of 73,728 data points covering a spectral width of 12,019 Hz were collected using 16 scans. The Free Induction Delay (FID) was zero-filled to 131,072 complex data points and an exponential window function was applied with a line broadening factor of 1.0 Hz prior to Fourier transformation. The spectra were automatically phase and baseline corrected.

#### Quality control, scaling and calibration of the NMR spectra

Further data processing was performed in Matlab® (R2009a; The Mathworks Inc., Natick, MA, USA) and described in Vaarhorst et al. [40]. In brief, the spectra and associated data were converted into Matlab files using in-house code. First, the spectra were combined into one file while removing superfluous information. For CPMG this included dropping the imaginary part of the spectrum, while for the JRES spectra the sum projection along the indirect dimension was taken. Quality control (QC) on the set of ^1^H-NMR spectra was carried out by examining a set of spectroscopic parameters such as shim values and intensity of the water signal, and subsequently visually inspecting the spectra. Spectra that failed the quality control were not included for further analysis. The remaining spectra were scaled with respect to the sensitivity of the receiver coil. This sensitivity is inversely proportional to the pulse length, which is dependent on the tuning of the RF coil. After subtracting a constant value as a simple baseline correction, the spectra were calibrated with respect to the anomeric resonance of α-D-glucose (δ = 5.23 ppm) [47]. Since there are small deviations of the signal position in the different ^1^H-NMR spectra, alignment was performed using the correlation optimized warping algorithm by Tomasi et al. [48]. This was performed actively for the CPMG spectra, after which the same warping was applied to the JRES projection. The peaks in the JRES projection were automatically deconvoluted by fitting the spectra with mixed Gauss-Lorentz line shapes using the Simplex method. As the fitting algorithm incidentally converges to a local minimum, values further from the median than three times the interquartile range were discarded. Using partial least square regression, the remaining signal intensities were used to build a linear model that predicts the intensities directly from the non-warped spectrum, yielding also reasonable values for the cases where the deconvolution or warping algorithms failed.

Finally, metabolites were assigned using information from the Human Metabolome Database (HMDB) and the Pearson correlation coefficients between the peak intensities [49].

### Statistical analyses and data processing

Student’s *t*-test and Chi-square tests were used to compare demographic characteristics between cases and controls. Raw ^1^H-NMR signal data were processed as follows. Values below [mean - 4 * SD] and above [mean + 4 * SD] were filtered out. Then normality was assessed and data were log_10_-transformed when necessary, using SPSS software version 20.0 (SPSS Inc., IBM, Armonk, NY, USA). Signal data was adjusted for kinship by linear regression in GenABEL version 1.7–0, using R version 2.14.2 (R Foundation for Statistical Computing, Vienna, Austria) [50]. Finally, the residuals from this linear regression model were transformed into Z-scores to approximate normality using SPSS software version 25.0 (SPSS Inc., IBM, Armonk, NY, USA). To reduce the dimensionality of the data and due to possible correlations between the parameters, elastic net regression was used to select a subset of the most informative signals for: (1) lifetime migraine diagnosis, and (2) a diagnosis of active migraine (defined as having at least one severe migraine in the last 12 months). Of note, patients likely had many attacks in the last year as is typical in migraine patients when they still have migraines, but data are lacking to assess how many attacks they had and when the last attack was before blood withdrawal nor do we know whether they were on medication. Hence we consider our migraine cases a sample with “real-life variation” with respect to attack frequency and severity. The R package glmnet was used with alpha set to 0.5 and 50-fold cross-validation using R software version 3.6.1 [51]. In this cross-validation step we validated the selection of the signals by performing our regression analysis on 50 randomly chosen samples of our study population. Elastic net regression reduces variance and error and increases bias and the predictive power, which leads to better long-term prediction. However, the inferential capability decreases, which makes interpretation difficult as there are no uncertainties in terms of confidence intervals or hypothesis testing.

In an attempt to interpret our findings, we performed subsequent regression models. Because we had to perform the regression models within the unique cohort the exact *p*-values of these models are no longer valid, although the results may provide at least some information whether metabolites may be involved. For the regression models we entered the metabolites of the metabolic profiles in a logistic regression model to determine the weights for each signal for this population. The linear predictor of the logistic regression model was used as a “weighted metabolite score” (sum of regression coefficients multiplied by the corresponding covariate values). This score was used in a second logistic regression analysis to calculate odds ratios (ORs), *p*-values and the proportion of explained variance. To determine whether we had to correct our logistic regression model we independently assessed the influence of sex, age, body mass index (BMI) and smoking status on the “weighted metabolite score”, by visually inspecting stratification plots and performing a linear model, where the “weighted metabolite score” was modelled as a function of migraine status. We included age, sex, BMI and current smoking status as covariates in the logistic regression model. To validate the findings from the previous analysis we performed analysis of variance (ANOVA) in which we compared the performance of the full model with the identified scores for migraine with the performance of a model containing only information on age, sex, BMI and smoking.

## Results

### Study population

**Fig. 1:**
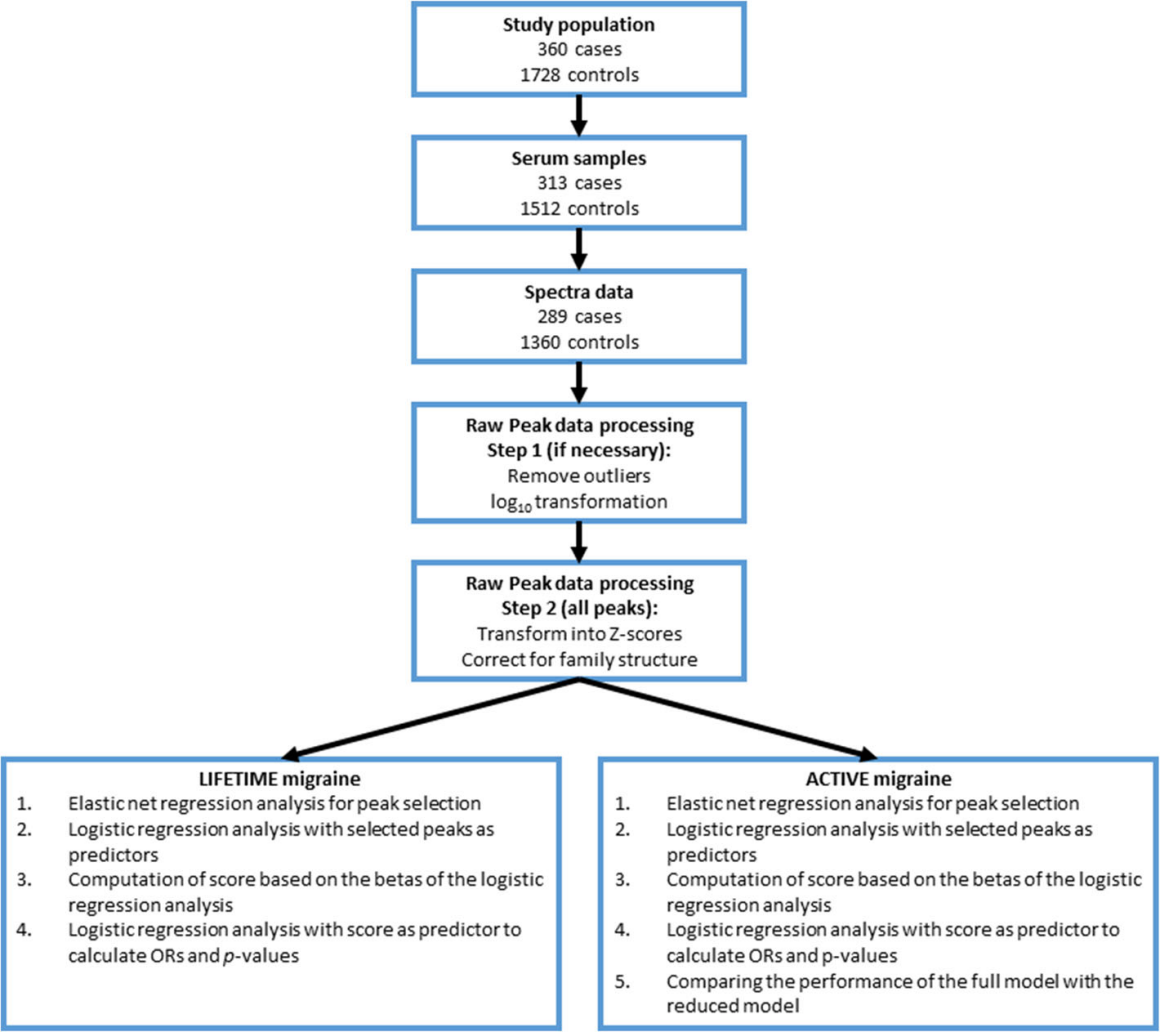
Flow chart of the patient flow and analysis steps

We conducted a case-control study with in the ERF population cohort and included 2088 participants in the study of which 360 were lifetime migraine patients and 1728 without severe headache served as controls. Eight-hour fasting serum samples were available from 313 migraine patients and 1512 controls that were used for ^1^H-NMR spectroscopy profiling (see Fig. 1).

### Signal detection, assignment and processing

A total of 100 metabolite signals were detected in the JRES projection and quantified in the ^1^H-NMR spectra [37]. For 82 signals, metabolites could be assigned. These 82 signals represented 49 different metabolites (See Additional file 1 for signal assignment). The other 18 signals could not be annotated. In total, good-quality ^1^H-NMR spectra were obtained from 289 migraine patients and 1360 controls. For 19 signals (out of 100) outliers were removed and nine signals were log_10_-transformed (See Additional file 1). The remaining data points of the 100 signals were used for the association analyses with migraine.

### Demographic characteristics

The demographic characteristics of the study population of whom good ^1^H-NMR data were obtained are shown in Table 1. Migraine patients tended to be younger (*p* = 0.013) and more often were female than controls (*p* <  0.001). In addition, lifetime migraine patients more often than controls were smokers (44.6% cases vs. 35.1% controls *p* = 0.006). No difference in BMI was observed between cases and controls (*p* = 0.934). Of the 289 lifetime migraine patients, 150 (52%) reported at least one severe migraine attack in the 12 months preceding the interview and were assigned to the group of “active migraine patients”. The active migraine patients consisted of 124 women (83%), had a mean age of 44 (SD ± 11.4), 71 (47.3%) were currently smoking and had a mean BMI of 26 (SD ± 4.9). Next, we assessed the influence of age, sex, BMI and smoking (see Additional file 2) on the weighted metabolite score. All covariates showed to be of influence on the weighted metabolite score and were added to the logistic regression model.

**Table 1:** Demographic characteristics

Variable	Lifetime migraine patients (***N*** = 289)	Controls (***N*** = 1360)	***p***-value	Active migraine patients^**b**^ (***N*** = 150)	***p***-value
Age (years)	46.5 ± 12.1	48.7 ± 14.5	0.013^c^*	44.0 ± 11.4	< 0.001^c^*
Female sex (%)	220 (76.1)	673 (49.5)	< 0.001^d^*	124 (82.7)	< 0.001^d^*
BMI	26.9 ± 5.0	26.8 ± 4.6	0.934^c^	26.3 ± 4.9	0.219^c^
Smoking^a^ (yes) (%)	129 (44.9)	481 (36.0)	0.006^d^*	71 (47.3)	0.008^d^*
MO patients	163 (56.4)	–	–	77 (51.3)	–

Values are expressed as absolute values and percentage or mean ± SD. Numbers and proportions may not add up to total of 100 due to rounding or missing values; ^a^Defined as currently cigarette smoking; ^b^Defined as having at least one severe migraine attack in the last 12 months; ^c^Student’s *t*-test; ^d^Chi-square Test; *Significant *p*-values (*p* < 0.05). Missing values in lifetime migraine patients for BMI (*n* = 2), smoking status (*n* = 2) and in controls for BMI (*n* = 24) smoking status (*n* = 27). MO = migraine without aura, BMI =  body mass index

### Association of metabolites with lifetime migraine diagnosis

Elastic net regression analysis of all 289 migraine patients and 1360 controls for all 100 signals identified six ^1^H-NMR signals as the best prediction subset. These signals were representative of four different metabolites (isoleucine, methionine, 1,5-anhydrosorbitol and creatine) and one unknown signal (Table 2). Subsequent logistic regression analysis showed support for association (odds ratio (OR) = 2.72; 95% confidence interval (CI) 1.97–3.75; *p* = 1.28 × 10^− 9^) explaining 3.9% of the variance in migraine status (Nagelkerke R^2^). After correction for age, sex, BMI and smoking the association no longer showed support for association (OR = 1.49; 95% CI 0.99–2.23; *p* = 0.051).

**Table 2:** ^1^H-NMR signals associated with lifetime migraine patients and active migraine patients

***Lifetime migraine patients***		***Active migraine patients***	
Metabolite	Chemical shift (ppm)	Metabolite	Chemical shift (ppm)
Isoleucine	0.92847	Cholesterol	0.89006
Isoleucine	0.99919	Isoleucine	0.92847
Unknown	1.40660	Unknown	0.95118
Methionine	2.63742	Leucine	0.95702
1,5-Anhydrosorbitol	3.58832	Isoleucine	0.99919
Creatine	3.92001	Lipids (CH2)^†^	1.26482
		Unknown	1.40660
		Acetate	1.90859
		Lipids (CH*2CH=CH)^†^	2.22215
		Pyruvic acid	2.36196
		Methionine	2.63742
		Dimethylglycine	2.91618
		Unknown	3.35396
		1,5-Anhydrosorbitol	3.58832
		Valine	3.59782
		Myoinositol	3.62232
		Glucose	3.72103
		Serine	3.95567
		Creatinine	4.04386
		Proline	4.12106
		Unknown	4.50117
		Glucose	5.22921

*Ppm* parts per million; ^†^The term in parenthesis indicates the structural feature of the lipids measured by ^1^H-NMR spectroscopy

### Association of metabolites with active migraine diagnosis

Next, we performed an elastic net regression on all 150 active migraine patients and 1360 controls for all 100 signals. This analysis identified 22 predictive signals. The subsequent logistic regression analysis was performed on 146 cases and 1343 controls, as not all subjects had sufficient signal data for all 22 signals. The regression showed support for association between the signal data and active migraine status (OR = 2.72; 95% CI 2.09–3.54; *p* = 1.35 × 10^− 13^) explaining 8.5% of the variance, this association remained after correction for sex, age, BMI and current smoking status (OR = 1.84; 95% CI 1.34–2.53; *p* = 1.64 × 10^− 4^) with a total explained variance of 12.3% (Nagelkerke R^2^). Hosmer and Lemeshow shows a good fit of the final model (*p* = 0.688). The outcome of our ANOVA analysis (*p* = 7.1 × 10^− 5^) added to the evidence for involvement of these metabolites in active migraine patients. The majority of the 22 signals have been annotated to metabolites, but four remained unknown (Table 2). The known metabolites that were relevant to distinguish metabolic profile of migraine patients from controls were cholesterol, isoleucine, leucine, lipids (CH2 and CH*2CH=CH), acetate, pyruvate, methionine, dimethylglycine, 1,5-anhydrosorbitol, valine, myoinositol, glucose, serine, creatinine, and proline. Our data suggests that there is a metabolic profile for active migraine that distinguishes them from controls even after correcting for age, sex, BMI and smoking status. Remarkable is that five of the six signals predictive for lifetime migraine status are also predictive for active migraine status (Table 2).

## Discussion

Here we investigated metabolites identified by ^1^H-NMR spectroscopy in serum of migraine patients and controls to assess whether metabolic profiles can distinguish the two groups. We identified 22 metabolites that were predictive for active migraine and estimated that they would remain predictive after correction for age, sex, BMI and smoking status. Active migraine status was linked with metabolic profiles with more (22) metabolites, when compared with lifetime migraine (6), suggesting that active migraine patients may have a more disturbed metabolic profile compared to lifetime migraine patients, at least among the 100 measured metabolites. Although, based on our study, it is not possible to directly interpret the *p*-values nor to make association on an individual metabolite level among the total 22 compounds associated with active migraine, it is remarkable that the majority of these 22 metabolites have been (indirectly) implicated in migraine before. In our study, we found metabolites involved in lipid metabolism namely; cholesterol, and two types of lipids (CH2 and CH*2CH=CH). A number of studies have previously implicated lipid metabolism in migraine for instance, epidemiologic studies have shown that obesity is a risk factor for migraine and that there is a comorbidity of cerebrovascular and cardiovascular disease and migraine [52, 53]. Some studies found an elevated total cholesterol, LDL-cholesterol, or triglycerides, and decreased levels of HDL-cholesterol in migraine [54, 55], whereas several other studies found no significant differences in lipid profiles [54, 56]. A recent meta-analysis encompassing 2800 migraine patients and 7353 controls from eight Dutch cohorts, using a different ^1^H-NMR metabolomics platform in a systematic approach, also showed alterations in HDL metabolism, in that study defined by a decreased level of lipoprotein A1 and a decreased free cholesterol to total lipid ratio in small HDL subspecies [55]. Neurovascular and endothelial dysfunction are believed to be an underlying cause for the increased risk in cerebrovascular and cardiovascular diseases in migraine patients [57, 58]. At the basis of this involvement lies a possible higher prevalence of risk factors, such as hypertension and hyperlipidaemia, in migraine patients [57]. Also the involvement of lipids in migraine pathophysiology has been shown in various studies [54, 55]. Regardless, the exact role lipids play is complex and needs further investigation.

Glucose is another metabolite we found that has previously been associated with migraine either directly or via metabolically associated pathways. Glucose levels and insulin metabolism, as well as mitochondrial dysfunction have been known to play a role in migraine pathology [59, 60]. Still, no association was found between migraine and diabetes type 2 [59, 61, 62]. It has been suggested that outside attacks, migraine patients have an impaired insulin sensitivity and higher fasting plasma insulin levels compared to controls [63, 64]. Recently it was shown that glucose levels were higher during a spontaneous migraine attack compared to outside of an attack [65]. Both 1,5-anhydrosorbitol and myoinositol, which were part of our prediction model, are involved in glucose metabolism. 1,5-Anhydrosorbitol is a naturally occurring monosaccharide found in nearly all foods and myoinositol, which is a carbocyclic sugar that is abundant in brain and other mammalian tissues, is synthesized from glucose 6-phosphate. Pyruvate is the conjugate base of pyruvic acid and is a key intermediate in several metabolic pathways throughout the cell. Pyruvic acid can be produced from glucose through glycolysis and it can supply energy to the cell via the Krebs cycle in the mitochondria. One study has investigated the lactic and pyruvic acid levels in the plasma of the migraine patients it was shown that both were significantly higher in migraine patients than in normal controls [66].

In addition, multiple amino acids were part of our prediction model for active migraine status, namely leucine, isoleucine, methionine, valine, proline and serine. Over the last decades, multiple amino acids have been hypothesized to play a role in migraine pathophysiology [19]. Leucine, isoleucine and valine are branched-chain amino acids (BCAAs), BCAAs have emerged as potential biomarkers of disease as they are associated with risk of cardiovascular disease, end-stage renal failure, and ischemic stroke [67]. In a small study of 37 migraine patients and 40 controls elevated levels of isoleucine in blood serum were found [68]. A recent study investigated amine pathways in 20 patients with migraine without aura, and 20 healthy subjects in serum with liquid chromatography coupled to mass spectrometry (LC-MS) [69]. This LC-MS study found decreased levels of leucine, isoleucine and methionine in migraine patients compared to controls. The valine, proline and serine concentration was not assessed directly in this study [69]. Although glutamate/glutamine has been repeatedly linked to migraine [70, 71], in our study the levels of glutamine/glutamate were not part of the predictive profile for migraine status.

As far as we know the other metabolites we found to be associated with active migraine status (acetate, dimethylglycine and creatinine) have thus far not been associated with migraine. Acetate is a monocarboxylic acid anion, which is metabolized mostly in peripheral tissues. Dimethylglycine, which is a derivative of the amino acid glycine, but it can also be a by-product of the metabolism of choline. Dimethylglycine has been suggested as a treatment for mitochondrial diseases [72] and in that sense might be associated with the migraine-glucose dysregulation. Creatine is involved in the conversion from adenosine diphosphate (ADP) back to ATP in muscle and is synthesized mainly in the liver from amino acids glycine and arginine.

We here identified a metabolite profile predictive for active migraine, a finding supported by the observation that several of its metabolites have already been reported in literature to be individually (in) directly associated with migraine. We would like to emphasize again that the focus of this study was to explore whether metabolite profiles can be linked to migraine status and less to show direct clinical relevance of individual metabolites.

A limitation of our study is that, the set of metabolites we studied using our metabolic profiling method covers only a small part of the human metabolome. Future, complementary, studies using different, more advanced, platforms may identify additional metabolites associated with migraine status. Additionally, in our study population we know to what extent patients are related and have the opportunity to correct for this. Future studies have to show to what extent our findings are also applicable to well-selected groups of migraine patients, for instance, with respect to frequency of attacks, time of last attack to blood withdrawal, possible comorbidities, etc. Another possible limitation is that in our study, model selection by elastic net regression was used for predictor selection to eliminate high correlations among predictors. This might lead to reduced transferability of prediction models, because correlation structures of predictors can vary between studies. Although we corrected for age, sex, BMI and smoking in our analysis, we cannot exclude a residual confounding effect of this variable nor of any other variable that we have not tested. Another limitation is that we used the same population for discovery of the associated signals and for assessing the magnitude of the association. Ideally, a replication study, to validate our findings, should be performed.

## Conclusions

In conclusion, using hypothesis-free metabolic profiling, by measuring a large set of metabolites using ^1^H-NMR spectroscopy, we identified a metabolomic profile consisting of 22 metabolite signals (lipids, amino acids and metabolites of glucose metabolism) that was predictive for active migraine status.

## Supplementary Information

**Additional file 1.** File with an overview of signals identified in the 2-dimensional J-resolved ^1^H-NMR spectrum and performed transformations

**Additional file 2.** File with ratio stratification plots for sex, age BMI and smoking status in lifetime migraine patients and active migraine patients

## Data Availability

The data used and/or analysed during the current study are available from the corresponding author on reasonable request.

## Abbreviations

^1^H-NMR: Proton nuclear magnetic resonance spectroscopy
ERF: Erasmus Rucphen Family
JRES: J-resolved spectra
CPMG: Purcell-Meiboom-Gill
OR: Odds ratio
CI: Confidence interval
CGRP: Calcitonin gene-related peptide
