# Supplementary material for: Metabolic profile changes in serum of migraine patients detected using 1H-NMR spectroscopy
Source: J Headache Pain. 2021 Nov 24;22(1):142. doi: 10.1186/s10194-021-01357-w (PMC8903680; doi:10.1186/s10194-021-01357-w)
Supplement: Supplementary file 1 — Additional file 1. File with an overview of signals identified in the 2-dimensional J-resolved 1H-NMR spectrum and performed transformations [file 10194_2021_1357_MOESM1_ESM.docx]

**Additional file 1.**

**Table 1.** Overview of signals identified in the 2-demensional J-resolved ^1^H-NMR spectrum and performed transformations

| **Signal number** | **Chemical shift (ppm)** | **Signal Assignment** | **Stat. Proc.** | **Signal number** | **Chemical shift (ppm)** | **Signal Assignment** | **Stat. Proc.** |
| --- | --- | --- | --- | --- | --- | --- | --- |
| 1 | 0.85113 | Unknown | none | 51 | 2.93035 | Asparagine | none |
| 2 | 0.87072 | Lipids (CH3) | LN | 52 | 3.02340 | Lysine | none |
| 3 | 0.89006 | Cholesterol | LN | 53 | 3.03376 | Creatine/Creatinine | none |
| 4 | 0.92847 | Isoleucine | none | 54 | 3.04941 | Ornithine | none |
| 5 | 0.94597 | Leucine | none | 55 | 3.14304 | Unknown | SD |
| 6 | 0.95118 | Unknown | none | 56 | 3.20231 | Choline | none |
| 7 | 0.95702 | Leucine | none | 57 | 3.21429 | Phosphorylcholine | none |
| 8 | 0.97200 | Unknown | none | 58 | 3.24004 | Glucose | none |
| 9 | 0.98082 | Valine | none | 59 | 3.26710 | 1,5-Anhydrosorbitol | none |
| 10 | 0.98612 | Unknown | none | 60 | 3.33236 | Proline | LN |
| 11 | 0.99919 | Isoleucine | none | 61 | 3.34504 | 1,5-Anhydrosorbitol | none |
| 12 | 1.03145 | Valine | none | 62 | 3.35396 | Unknown | none |
| 13 | 1.06258 | Unknown | none | 63 | 3.38052 | Unknown | none |
| 14 | 1.11139 | Ketoisovalerate | none | 64 | 3.40064 | Glucose | none |
| 15 | 1.13461 | Unknown | SD | 65 | 3.48648 | Glucose | none |
| 16 | 1.16301 | Isopropyl alcohol | SD | 66 | 3.53111 | Glucose | none |
| 17 | 1.17437 | Ethanol | SD | 67 | 3.55110 | Glycine | none |
| 18 | 1.19057 | 3-Hydroxybutyrate | SD+LN | 68 | 3.55914 | Glycerol | none |
| 19 | 1.21117 | Unknown | none | 69 | 3.58832 | 1,5-Anhydrosorbitol | none |
| 20 | 1.26482 | Lipids (CH2)^†^ | LN | 70 | 3.59782 | Valine | none |
| 21 | 1.31981 | Lactate | none | 71 | 3.62232 | Myoinositol | SD |
| 22 | 1.38997 | Unknown | none | 72 | 3.65021 | Ethanol | SD+LN |
| 23 | 1.40660 | Unknown | none | 73 | 3.65855 | Isoleucine | none |
| 24 | 1.42426 | Unknown | none | 74 | 3.71204 | Glucose | none |
| 25 | 1.47045 | Alanine | none | 75 | 3.72103 | Glucose | none |
| 26 | 1.70571 | Unknown | none | 76 | 3.74475 | Unknown | none |
| 27 | 1.90859 | Acetate | SD | 77 | 3.75932 | Glucose | none |
| 28 | 1.99964 | Lipids (CH*2CH=CH)^†^ | none | 78 | 3.77643 | Alanine | none |
| 29 | 2.03401 | N-acetyl glycoproteins | none | 79 | 3.80094 | Glucose | none |
| 30 | 2.06653 | O-acetyl glycoproteins | none | 80 | 3.81746 | Unknown | none |
| 31 | 2.10168 | Glutamine/Glutamate | none | 81 | 3.82382 | Glucose | SD |
| 32 | 2.11814 | Glutamine/Glutamate | none | 82 | 3.83140 | Unknown | none |
| 33 | 2.13291 | Acetylcarnitine | none | 83 | 3.83856 | Glucose | SD |
| 34 | 2.22215 | Lipids (CH2CO)^†^ | LN | 84 | 3.87709 | 1,5-Anhydrosorbitol | none |
| 35 | 2.26037 | Valine | none | 85 | 3.89397 | Glucose | none |
| 36 | 2.27276 | Acetoacetate | SD | 86 | 3.92001 | Creatine | none |
| 37 | 2.30052 | 3-Hydroxybutyrate | SD | 87 | 3.93298 | Unknown | none |
| 38 | 2.34857 | Glutamate | SD | 88 | 3.95567 | Serine | none |
| 39 | 2.36196 | Pyruvate | none | 89 | 3.97538 | Phenylalanine/Histidine | none |
| 40 | 2.39235 | 3-Hydroxybutyrate | SD | 90 | 4.04386 | Creatinine | none |
| 41 | 2.42815 | Glutamine | none | 91 | 4.10334 | Lactate | LN |
| 42 | 2.44561 | Glutamine | none | 92 | 4.12106 | Proline | none |
| 43 | 2.46282 | Glutamine | none | 93 | 4.23715 | Threonine | none |
| 44 | 2.52733 | Citrate | SD | 94 | 4.50117 | Unknown | SD |
| 45 | 2.59803 | Unknown | none | 95 | 5.17855 | Mannose | none |
| 46 | 2.63742 | Methionine | none | 96 | 5.22921 | Glucose | SD |
| 47 | 2.66908 | Citrate | SD | 97 | 5.29802 | Lipids (CH=CH)^†^ | LN |
| 48 | 2.70842 | Dimethylamine | none | 98 | 6.89014 | Tyrosine | none |
| 49 | 2.89562 | Unknown | none | 99 | 7.18628 | Tyrosine | none |
| 50 | 2.91618 | Dimethylglycine | SD | 100 | 8.44976 | Formate | none |

Ppm: parts per million; Stat. Proc.: Statistical Processing; LN: log transformation performed; SD: outliers > 4 standard deviations from the mean removed; † The term in parenthesis indicates the structural feature of the lipid measured by ^1^H-NMR spectroscopy.
