# Supplementary figures and images for: Metabolic profile changes in serum of migraine patients detected using 1H-NMR spectroscopy
Source: J Headache Pain. 2021 Nov 24;22(1):142. doi: 10.1186/s10194-021-01357-w (PMC8903680; doi:10.1186/s10194-021-01357-w)

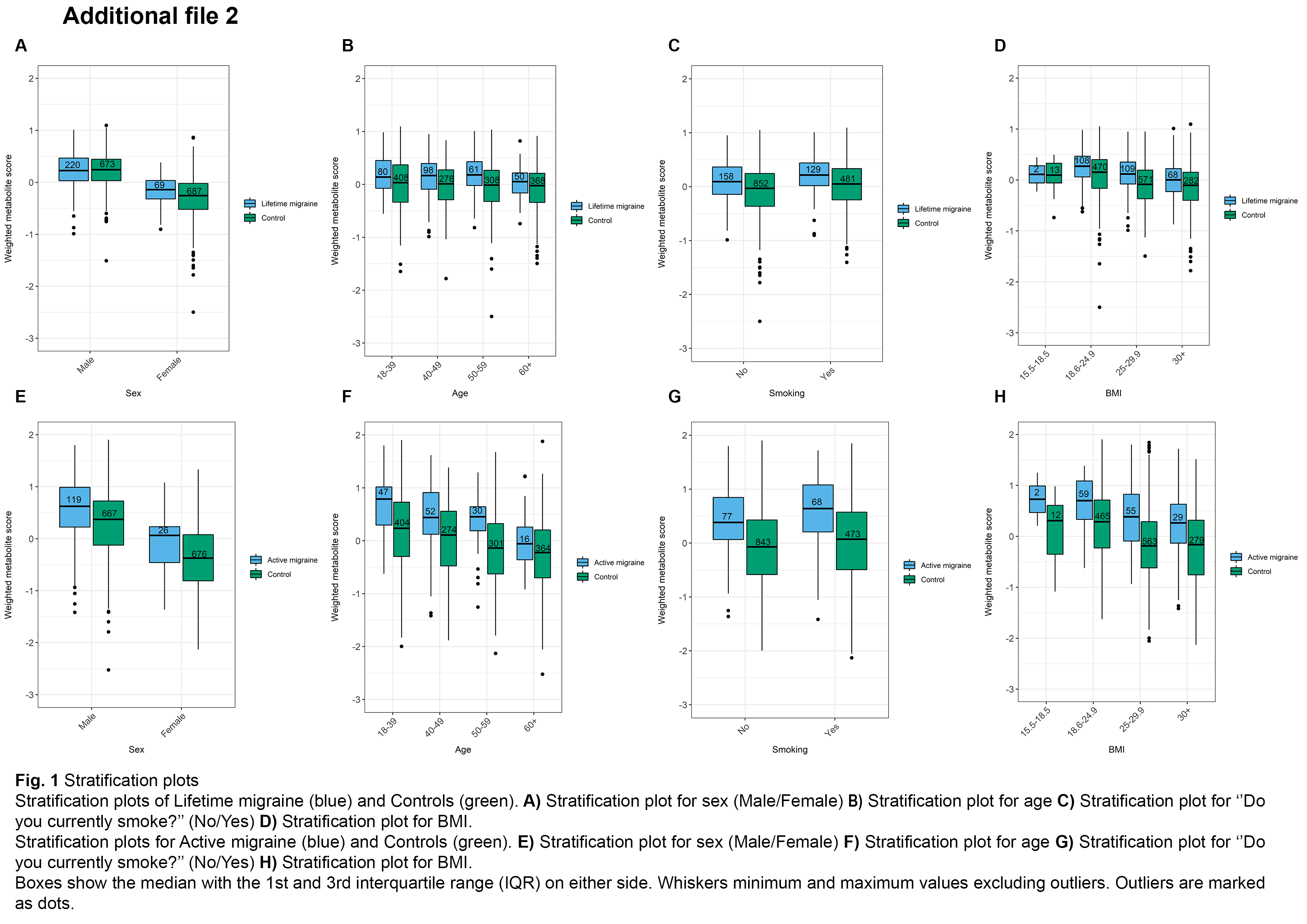

Supplement: Supplementary file 2 — Additional file 2. File with ratio stratification plots for sex, age BMI and smoking status in lifetime migraine patients and active migraine patients [file 10194_2021_1357_MOESM2_ESM.png]
